# Factors Associated with Healthy Aging among Older Persons in Northeastern Thailand

**DOI:** 10.1007/s10823-016-9296-y

**Published:** 2016-07-18

**Authors:** Pornpun Manasatchakun, Pleumjit Chotiga, Jacek Hochwälder, Åsa Roxberg, Maria Sandborgh, Margareta Asp

**Affiliations:** 1 School of Health, Care and Social Welfare, Mälardalen University, Box 325, Drottninggatan 16A, 63105 Eskilstuna, Sweden; 2 Boromarajonani College of Nursing Udon Thani, Udon Thani, Thailand; 3 Boromarajonani College of Nursing Chiang Mai, Chiang Mai, Thailand; 4 School of Health and Welfare, Halmstad University, Halmstad, Sweden; 5 VID Specialized University, Bergen, Norway

**Keywords:** Cross-sectional study, Healthy aging, Northeastern Thailand, Older persons

## Abstract

The aim of this study was to describe factors associated with perceived health and healthy aging among older people in northeastern Thailand. Thailand’s aging population is growing and facing an increasing old-age dependency ratio. Northeastern Thailand, known as Isan, is a region in which the number of older residents is projected to grow rapidly. Older people in this region are likely to confront great threats to their health and well-being. These issues require appropriate attention and actions to promote healthy aging. However, healthy aging in this region has not been studied. A cross-sectional study was conducted on a sample of 453 older people, aged 60 years or older. Participants completed the Healthy Aging Instrument (HAI) and provided relevant demographic characteristics. Mann-Whitney U tests, Kruskal-Wallis tests and multiple regression models were used to analyze the data. Through comparative analyses, significant differences in HAI scores were observed for the following factors: marital status, residential area, disability, income level, and perceived meaningfulness in life. In the multiple regression models, residential area, disability, and marital status explained 24.30 % of the variance in HAI scores. Health promotion strategies and future targeted intervention programs should consider the importance of these factors.

## Introduction

Thailand’s older population represents the second fastest growing group of people over 60 years in Southeast Asia (Fujioka and Thangphet 2009). The older population in Thailand stood at approximately 10 million (Knodel et al. 2013) and 15.80 % of the total population in 2015 (United Nations 2015). The number of older people is expected to reach to over 20 million or 30 % of the total population by 2035 (Knodel et al. 2015). The population of people aged 80 years and older, referred to as the “oldest old”, is estimated to increase from 10.98 % of the older population in 2020 to 12.15 % by 2030 (Sasat and Bowers 2013). Moreover, the mean predicted lifespan is expected to increase from 74 years in 2013 (World Bank 2015) to 79 years by 2050 (UNFPA 2006). This increase in age may contribute to the risk of chronic disease and disability, which will have an impact on older people’s health (Westaway 2010).

### Social Welfare and Policies for Older People in Thailand

Thailand’s government is aware of the aging situation and intends to develop policies to support older persons. The Second National Plan for Older Persons, 2002–2021 is a policy established to respond to and serve the growing number of older persons. The key domains of this plan are activities to improve older person’s well-being and quality of life, e.g., supporting long-term care, promoting positive attitudes toward older persons, and protecting their rights (Jitapunkul and Wivatvanit 2009; Sasat and Bowers 2013). According to this plan, healthy aging is one strategy to ensure a good quality of life among older people (Jitapunkul and Wivatvanit 2009). In addition, the government provides health care schemes such as *Compulsory Health Insurance,* which covers formal employees in the private sector and the *Civil Servant Medical Benefit Scheme,* which provides benefits for government officers and their families to allow members of both the active and retired populations to access health care services (Sasat and Bowers 2013). Further, *Universal Health Insurance Coverage* was promoted for older persons with no support from the government or private health insurance to ensure that they receive free health care services from the public sector (Jitapunkul and Wivatvanit 2009; Sasat and Bowers 2013). To decrease poverty in old age, social welfare assistance helps poor older persons and those who do not receive permanent income from public enterprises, government, and local business through monthly public pensions. This type of pension is related to a person’s age (Sasat and Bowers 2013). Older persons who receive social welfare assistance receive monthly allowances of 600 baht (U.S. $18), 700 baht (U.S. $ 21), 800 baht (U.S. $24), and 1000 baht (U.S. $30) for those who aged 60–69, 70–79, 80–89 and 90 or older, respectively (Suwanrada 2012). Although older people can use these pensions to meet their basic needs, the amount of money is insufficient, especially in the absence of other incomes (Sasat and Bowers 2013). Some recipients also have to share their benefits with members in their household.

#### The Isan Region

Thailand is divided into four regions: central, northern, northeastern, and southern. The aged population has never increased at a uniform rate throughout the country. It is important to note these regional variations when formulating policies and allocating resources to address issues relating to the aging population. In northeastern Thailand (Isan), the number of older people is increasing more than in other regions, and lifespans are clearly extending (UNFPA 2006). Geographically, Isan is the largest region and contains one-third of the population of Thailand (Mongkolsawat et al. 2001). The older population in this region represents over one-third of its overall population (Knodel & Chayovan 2008). The majority of Isan people are involved in agriculture (Ekasingh et al. 2007). These people have always suffered from poverty (Caffrey 1992; Fry and Kempner 1996). Isan has poorer natural resources than other regions in Thailand (Fry and Kempner 1996). According to the Word Bank’s report (World Bank 2005), the poorest population lives in this region.

Despite Isan’s poverty, this region has a rich culture (Fry and Kempner 1996). Isan culture is closely related to Lao, where Isan’s ancestors originated (Kutanan et al. 2014). Most people in Isan, like people in other regions of Thailand, are Theravada Buddhist (Hayashi 2003). The central doctrine of Theravada Buddhism is the *Four Noble Truths*: life is unsatisfactory, there are causes of this dissatisfaction, there is an end to suffering and the way out of all disappointment and suffering is the Eightfold Path (Kalab 1990; Ratanakul 2007). Buddhists believe in the law of *Karma*, which asserts that every life situation can be explained as a result of a cause (Klunklin et al. 2005). Buddhism has been a part of the cultural background that influences Isan peoples’ beliefs, thoughts, and attitudes (Wongtham et al. 2015). In Isan culture, monks play a key role in influencing the morals of the people (Wongtham et al. 2015). One moral principle that is prescribed by Buddhism is parent repayment, which is perceived as a highly valued virtue (Choowattanapakorn 1999). Children are expected to care for and financially support their parents to repay them for raising them. This reciprocal relationship is predominant in the Buddhist doctrine.

Isan people practice unique traditions that they have strictly followed since ancient times. One such tradition is called *Heet Sib Song – Klong Sib See* (Lawalee et al. 2014; Wanlu et al. 2009). This tradition is a moral standard in a society that is based on the principles of Buddhism (Wanlu et al. 2009). The main component of *Heet Sib Song – Klong Sib See* is paying respect to older people through a religious ceremony (Wanlu et al. 2009). Aside from this ceremony, traditional Isan literature influences people’s lifestyles and teaches children to take responsibility for their roles and exhibit thankfulness toward their ancestors (Sansak et al. 2014).

Societal changes have altered the Isan lifestyle from its original form. These changes include changing family relationships and changing patterns of family structure because of successful family planning policies in Thailand (UNFPA 2006). In this case, older persons who have no children may depend on others for support and for help to meet their physical and psychological needs (Knodel and Chayovan 2008). Furthermore, young people in the Isan region tend to immigrate from their hometowns to cities to take advantage of modern culture and to try to escape poverty, which may limit opportunities to live with and care for their parents. Older persons in this region may feel abandoned by the younger generations (Sudnongbua et al. 2010). Therefore, aging constitutes a major challenge for healthcare professionals who plan strategies to promote health and sustain the well-being of older persons in the Isan region.

#### Healthy Aging and Perceived Health

Healthy aging is an important determinant for improving and preserving health and wellness among older people in Thailand (Thiamwong et al. 2013). Although healthy aging is adopted as a national policy to promote the health of the older population (Jitapunkul and Wivatvanit 2009; Williamson 2015), few studies have addressed healthy aging. Some studies (Danyuthasilpe et al. 2009; Thiamwong 2008; Thiamwong et al. 2013) have focused on the definition and the process of healthy aging in qualitative studies conducted from the perspectives of older persons. One qualitative study (Thanakwang et al. 2012) considers how the factors that contribute to healthy aging have evolved. Further, studies have shown that healthy aging is influenced by culture (Danyuthasilpe et al. 2009; Thanakwang et al. 2012). However, no recent studies have referred to factors associated with healthy aging in the Isan culture.

In this study, healthy aging refers to a multidimensional concept involving a variety of physical, psychological, sociocultural, and spiritual factors (Thiamwong 2008). This construct has been operationalized in the healthy aging instrument (HAI) (Thiamwong 2008; Thiamwong et al. 2008). A healthy ager is “a Thai older adult who describes himself or herself as healthy, but not necessarily free from chronic diseases or disability” (Thiamwong 2008, p.7). This description represents perceived health, which refers to peoples’ subjective experiences, i.e., how they understand and interpret their health. By definition, healthy aging (Thiamwong 2008) is related to perceived health.

#### The Main Determinants of Perceived Health and Healthy Aging

A conceptual framework of factors associated with perceived health and healthy aging was identified based on a review of the existing studies (Benyamini et al. 2000; Çapik and Bahar 2008; Cott et al. 1999; Faresjö and Rahmqvist 2010; Molarius and Janson 2002; Ren 1997; Singh et al. 2013; Skrabski et al. 2005; Thanakwang 2009; Wen et al. 2006; Zunzunegui et al. 2004). The following factors were identified as pertinent to perceived health: socio-demographic factors, health status factors, and perceived meaningfulness in life.

##### Socio-Demographic Factors

The area of residence was a key factor for perceived health (Thanakwang 2009). Perceived health decreased among people who lived in rural areas (Monnat and Pickett 2011) and significantly increased with chronological age (Thanakwang 2009). Gender has been associated with differences in perceived health (Singh et al. 2013). Women perceived their health to be worse than that of men (Undén et al. 2008). High income (Çapik and Bahar 2008), high education levels (Faresjö and Rahmqvist 2010), being married (Ren 1997), and individual social support (Thanakwang 2009; Wen et al. 2006; Zunzunegui et al. 2004) appear to highly influence individual health perceptions.

##### Health Status Factors

The association between health status and perceived health are fairly consistent. Disease (Molarius and Janson 2002) and disability (Cott et al. 1999) influence individuals’ perceived health in a negative way.

##### Perceived Meaningfulness in Life

People perceive their health as related to what they consider meaningful, and perceived meaningfulness in life is positively correlated to health (Skrabski et al. 2005).

Several factors have been identified in the literature that appear to be associated with perceived health. According to this review of empirical studies, the following were factors of interest: residential area, gender, education level, income level, age, marital status, underlying disease, disability, social support from family and healthcare provider, and perceived meaningfulness in life. An improved understanding of these factors and their associations with perceived health and healthy aging in the Isan-Thai culture could help healthcare professionals or public health officers design effective programs to improve health care and promote healthy aging in this region.

### Aim

The aim of this study was to describe factors associated with perceived health and healthy aging among older people in northeastern Thailand.

## Materials and Methods

### Design

The study had a cross-sectional design.

### Setting and Participants

The study was conducted in northeastern Thailand, which includes 20 provinces. The participants were older adults living in the Udon Thani province (*N* = 159,688), which is located approximately 564 km from Bangkok. This province was selected as the study site because it ranks among the top ten provinces in the Isan region with a population of adults aged 60 and older (Institute for Population and Social Research Mahidol University 2014). Furthermore, this province has experienced rapid urbanization and lifestyle changes because of this urbanization (Phuttharak and Dhiravisit 2014). According to the public administration, the Udon Thani province is divided into separate districts (Amphoes), which are in turn divided into 5 levels. The level assigned to a district depends on the size of the population; the revenue collected from taxes; and the number of district facilities, social services and government offices.

The sampling method consisted of six stages. First, 20 districts were separated into 5 subcategories based on the level assigned (first, second, third, fourth, and fifth levels) using the cluster method. Then, one district per level was selected via simple random sampling. Five of the twenty districts were selected in the first sampling stage. Each district was then divided into municipal and non-municipal areas based on their administrative classification. Next, five municipalities and five non-municipalities were selected via simple random sampling. Finally, both the municipalities and non-municipalities provided a list of all residents aged ≥60 years in each area, and these people were selected via stratified systematic sampling. An identification number was assigned to each participant. Inclusion criteria were age ≥ 60 years and ability to speak Thai. Exclusion criteria were psychiatric problems that might interfere with memory or judgment. This criterion was determined by the community health nurses based on the family folder. The response rate was 98.50 % or 453 out of 460 participants. Table 1 describes the final sample.

**Table 1:** Sample characteristics (*n* = 453)

Characteristic	n	%
Area of residence		
Inside the central city	100	22.10
Outside the central city	353	77.90
Gender		
Female	304	67.10
Male	149	32.90
Age (years)		
60–75	357	78.80
> 75	96	21.20
Marital status		
Married	255	56.29
Divorced/widowed	188	41.50
Unmarried	10	2.21
Education		
None	39	8.60
Primary education	401	88.50
Secondary education	13	2.90
Occupation		
None	17	3.75
Farmer	267	58.94
Former employed	147	32.45
Self-employed	22	4.86
Income		
None	33	7.30
≤ $167	285	62.90
$168–334	119	26.30
≥ $335	16	3.50
Underlying disease		
No	215	47.50
Yes	238	52.50
Types of disease**		
Diabetes	139	42.38
Hypertension	169	51.52
Other (gout and kidney disease)	20	6.10
Disability		
None	410	90.50
Yes	43	9.50
Type of disability		
Hearing impairment	16	37.21
Vision impairment	8	18.60
Physical disability	19	44.19
Living condition		
Alone	103	22.74
With children/spouse	350	77.26
Care from a family member		
No	84	18.54
Yes	369	81.46
Contact with healthcare provider		
No	4	0.83
Yes	449	99.17
Amount of perceived meaningfulness in life		
Less to moderate	82	18.10
High to very high	371	81.90

**Selected more than one item

### Measurements

A two-part questionnaire was used for data collection. The first part of the questionnaire addressed socio-demographic factors, health status and perceived meaningfulness in life. The second part was the HAI.

#### Part one: Socio-Demographic Factors

Socio-demographic factors included gender, age, marital status, area of residence, education level, income level, and social support. Three of these factors were dichotomous: gender, area of residence and social support. Gender was divided into female or male. Age was divided into two groups: young-old (60–75 years old) and old-old (> 75 years). Marital status was separated into three subgroups: married, divorced/widowed, or unmarried. Area of residence was divided into two subgroups: inside the central city of the province and outside the central city of the province. Education level was divided into three subgroups: none, primary education and secondary education. Income, from salary and/or retirement payments, was divided into four subgroups: none, low income (≤ $167 per month), middle income ($168–334 per month), and high income (≥ $335 per month). The questions concerning social support covered care from family members and contact with healthcare providers at a frequency of at least 1 time per month. Family members were defined as a spouse, children and other relatives. Healthcare providers were defined as physicians, dentists, pharmacists, and registered nurses. Care from family members was measured by the question, “Do you receive any care from your family?” Contact with healthcare providers was measured by the question “Do you have any contact with healthcare providers?” Both questions could be answered “Yes” or “No”.

#### Health Status

Health status included both disease and disability. Disease was defined as an underlying disease that had been diagnosed by a physician. Disease was measured as a dichotomous variable coded “Yes” or “No” using the question “Do you have any underlying diseases?” The participants could then select types of disease, e.g., diabetes, hypertension, gout, and kidney disease.

Disability was defined as impairment and measured as a dichotomous variable coded “Yes” or “No” using the question “Do you have any disabilities?” The types of disability could be described as, e.g., hearing impairment, vision impairment, or physical disability.

#### Perceived Meaningfulness in Life

Perceived meaningfulness in life was measured using the question “Do you feel that your life is meaningful?” Responses were rated on a 5-point Likert scale ranging from 1 (not at all) to 5 (to a very high degree).

#### Part two: the Healthy Aging Instrument (HAI)

The HAI consists of 35 items. Responses are provided on a 5-point Likert scale indicating how true certain statements are for participants, where 1 = absolutely not, 2 = less likely, 3 = not sure, 4 = more likely and 5 = absolutely yes. Total HAI scores range from 35 to 175, and higher scores indicate healthier aging persons (Thiamwong et al. 2008). The HAI includes 9 factors: self-sufficiency and living simply (e.g., “Everything I have is sufficient”), accepting aging (e.g., “I accept that I am not able to do things that I used to”), managing stress (e.g., “I do not worry without reason”), having social relationships and support (e.g., “My neighbors and I help each other”), making merit and good deeds (e.g., “I always do good deeds”), practicing self-care and self-awareness (e.g., “I am not worried that I am older”), staying physically active (e.g., “I feel weak if I do not do anything”), staying cognitively active (e.g., “I stay mentally active to prevent forgetfulness”) and participating socially (e.g., “I participate in community activities”). Five experts evaluated the HAI for its content validity, and it was agreed that this instrument is appropriate for use among older people in Thailand. The internal consistency of the total questionnaire in a sample of older people in south Thailand was acceptable: Cronbach’s alpha =0.88 (Thiamwong et al. 2008). To test face validity, a pilot study (*N* = 30) was conducted in the present study. The respondents were asked for feedback on each part of the questionnaire, and no problems or misunderstandings arose. The internal consistency of the total questionnaire for the pilot sample was high: Cronbach’s alpha =0.96.

### Procedure

The Board Committee of the Udon Thani Provincial Public Health Office of Thailand and the regional ethics committee, Uppsala Sweden approved this study (Dnr 2013/019). The first author selected three research assistants who were village health volunteers and were not in the research areas to guarantee that the participants were not dependent on the research assistants for care. The research assistants were chosen based on their educational background and research experience collecting data. The first author wrote guidelines for the research assistants and trained them to ensure their skills in data collection. The first author and the research assistants explained the information letter and described the purpose, procedure and possible benefits and risk to the participants. Furthermore, the first author and the research assistants explained that the participant’s confidentiality would be respected and that they were free to withdraw from the study at any time. The participants were given code numbers to protect their privacy. Older persons who chose to participate were asked to sign an informed consent form. The participants answered the questionnaires by themselves; however, if the older persons were unable to read or write, the first author or research assistants helped the participants to understand and complete the questionnaire. It took approximately 25 min to complete the questionnaires. The completed questionnaires were sent to the researchers.

### Data Analyses

Descriptive data are presented as numbers and percentages. The total HAI scores were compared for two subgroups using Mann-Whitney U tests and for three or more subgroups using Kruskal-Wallis tests. The significance level was set at *p* ≤ 0.05. Then, variables in bivariate and multivariate analyses that significantly differed by subgroup with regard to perceived health and healthy aging were selected to test individual predictors within a multiple regression model. A backward-selection multiple regression analysis was then conducted to test the factors that predicted perceived health and healthy aging. The significance level for the multiple regression analysis was set at *p* ≤ 0.05. The analysis was performed using SPSS version 17.0 (Statistical Package of the Social Sciences 2007).

## Results

The sample characteristics are described in Table 1. A majority of the sample was female, was married, lived outside the central city of the province, or had completed primary education. Some 52.50 % of the sample had an underlying disease, of which hypertension was the most commonly reported. Diabetes was the second-most commonly reported disease. Some of the participants reported some type of disability, of which physical disability was the most frequently reported. Just 3.50 % had a high monthly income. More than three-quarters of the participants lived with their children or spouse, whereas 22.74 % lived alone. Most of the respondents received care from a family member, e.g., children, or met a healthcare provider once a month. 81.90 % of the sample perceived that their life was meaningful high to very high degree. 

The mean HAI score was 145.25 (SD = 20.12), and the scores were normally distributed. Due to the small sizes of the compared subgroups, nonparametric statistics were used. The median HAI score was 148.38, with an interquartile range of 134–161. For the perceived meaningfulness in life factor, no participant responded “not at all”. Prior to analyses’ scores were dichotomized as 1 = less to moderate and 2 = high to very high meaningfulness in life. The HAI scores differed significantly among subgroups based on marital status, area of residence*,* disability, income level, and perceived meaningfulness in life. Single participants had the lowest HAI scores. The participants who lived in the central city of the province had significantly lower HAI scores, as did participants with disabilities. Furthermore, HAI scores were significantly lower among participants who did not have any income at all. The participants who reported having high to very high level of meaningfulness in life also had significantly high HAI scores. Tables 2 highlighted the differences among subgroups with regard to perceived health and healthy aging and present HAI scores that were reported for the subgroups.

**Table 2:** Subgroup HAI scores compared using the Mann-Whitney U test, Kruskal-Wallis test and post hoc analyses

Factor	n	Interquartile Range (25th, 75th percentile)	U	*p*-value	Post hoc analysis		
Area of residence			6721.50	0.00*	-	-	-
Inside the central city	100	(114, 141.75)					
Outside the central city	353	(142,162)					
Disability			4347.50	0.00*	-	-	-
None	410	(137, 161)					
Yes	43	(111, 144)					
Amount of perceived meaningfulness in life			12,036.00	0.03*	-	-	-
Less to moderate	82	(123.50, 158.25)					
High to very high	371	(137, 161 )					
Marital status			-	0.04*	Divorced/widowed† 228.23, 213.56† (*p* = 0.23)		Unmarried †
Married	255	(138,161)					135.18,77.30† (*p* = 0.02*)
Divorced/widowed	188	(131.25, 160.75)					101.48,62.30† (*p* = 0.04*)
Unmarried	10	(104.75, 152.75)					
Income level			-	0.00*	≤ $167†	$168–334†	≥ $335†
None	33	(108, 154)			85.12,168.11† (*p* = 0.00*)	59.89, 81.11† (*p* = 0.01*)	19.79, 35.75† (*p* = 0.00*)
≤ $167	285	(139, 161)				214.73,173.20† (*p* = 0.001*)	149.32,180.91† (*p* = 0.16)
$168–334	119	(132, 157)					64.94,90.78† (*p* = 0.01*)
≥ $335	16	(138, 167.75)					

* ***P*** **≤ 0.05; †Subgroup description**

In the backward-selection multiple regression model, the initial predictor variables were marital status, area of residence, disability, income and perceived meaningfulness in life. The HAI score was the dependent variable in this model. Table 3 describes the predictor variables. The final model included all entered predictor variables, of which three, area of residence, disability and marital status, were significant at *p* ≤ 0.05. The final model explained 24.30 % of the variance in HAI scores. However, income and perceived meaningfulness of life did not have a significant association with perceived health and healthy aging.

**Table 3:** Multiple regression analysis with HAI score as the dependent variable

Variable	B^a^	SE
Area of residence	16.06*	2.24
Marital status ^b^	3.80*	1.70
Disability	−12.87*	3.01
Income ^c^	6.43	3.46
Perceived meaningfulness in life	2.16	1.15
*Model summary*		
Model F	28.65	
df	452	
R^2^	0.243	

* ***P*** **≤ 0.05**

^a^B is the unstandardized regression coefficient

^b^Unmarried/divorced/widowed = 0 and married = 1

^c^no income = 0 and income = 1

## Discussion

The present study is the first to analyze factors associated with perceived health and healthy aging among older people in northeast Thailand. Three of the factors included in the regression model were associated with perceived health and healthy aging: area of residence, disability, and marital status.

### Residential Area, Disability, and Marital Status as the Key Factors

The analyses indicated that living outside central city was a major factor associated with perceived health and healthy aging. Our finding is inconsistent with a previous study (Thanakwang 2009) in which living in an urban area was a significant factor in perceived good health. Comparisons of perceived health and healthy aging based on area of residence, showed that older people who lived in the central city of the province had significantly lower HAI scores. This finding is not consistent with previous studies in both Western and Asian countries (Ahmad et al. 2005; Monnat and Pickett 2011), which claimed that people who live in rural areas were more likely to report having poor health than those who live in urban areas. For example, Monnat and Pickett (2011) explained that people in remote rural areas in the US have poor health due to structural disadvantages (Monnat and Pickett 2011). These findings may not apply to the northeastern region of Thailand. Although people in remote rural areas face disadvantages and may be influenced by inequality (Yiengprugsawan et al. 2007), the rural environment may be appropriate for old age. In Isan, Buddhism influences older people’s beliefs and lifestyles, especially those who live in rural area (Keyes 1984). Buddhism may give older people the opportunity for social interaction in the context of religious activities, stimulating good social relationships. Religious activities may be the source of good emotional support, which in turn is related to perceived health in a positive way. This finding suggests that healthcare providers should consider differences in residential areas that may contribute to the rural/urban disparities in health. Furthermore, future programs should provide appropriate support to older people who live in the central city of the province to promote healthy aging.

There was a negative correlations between disability and perceived health and healthy aging. This finding is in line with previous studies wherein functional disability was found to have a negative effect on perceived health (Benyamini et al. 2000; Jang et al. 2006). One might argue that when a person with a disability experiences a health problem, the disability can have an even greater effect on his/her life. When we compared older people’s perceived health and healthy aging based on disability, we found that older people with disabilities had significantly lower HAI scores than those without disabilities. This result matches that of a previous study (Cott et al. 1999) in which disability was associated with poor health. Regarding the characteristics of healthy aging based on the HAI, older persons who perceived themselves as a healthy are not necessarily free from chronic disease or disability (Thiamwong 2008). However, according to our results, disability is negatively correlated with perceived health and healthy aging. Disability could result in limitations in daily task performance or social activity participation due to impairments in vision, hearing, mobility, communication, cognition, emotion, behavior, intellect, or learning, which may influence a person’s perceived health. Based on our findings, it is suggested that in programs promoting healthy aging, older people with disabilities should be a first priority. Disability, therefore, should be considered more carefully by policy makers when improving the policies to promote healthy aging. Understanding the differences between older persons with disabilities and those without with regard to HAI scores may be important in designing interventions for older persons with disabilities.

Another socio-demographic factor associated with perceived health and healthy aging was being married. This finding is consistent with that of Ren (1997). One might argue that married persons’ support from their spouse or children is connected to positive effects on individual health (Gurung et al. 2003). When we compared the perceived health and healthy aging between subjects with various marital statuses, older people who were unmarried scored lower on HAI than others. This result is consistent with those of Palner and Mittelmark (2002), who reported that married people have a higher level of perceived mental health than unmarried people. Being married might confer health advantages that influence perceived health. These could be, e.g., emotional support, instrumental support, and social exchange with the partner (Goldman et al. 1995; Palner and Mittelmark 2002). However, we also found that older people who were divorced or widowed reported higher HAI scores than those who were unmarried, possibly because they receive emotional support from their children (Zunzunegui et al. 2004). This support may be related to moral principles of Buddhism and Isan traditions according to which children are expected to be the primary sources of support for older persons. They can help older people with personal management, physical care, economic, and emotional support. Based on the present findings, one suggestion is that healthcare providers or policy makers should target unmarried and childless older people to promote healthy aging. Because community support is considered important in the Isan culture (Rukwong 2008), another suggestion is therefore that neighbors or friends of older people should be involved in promoting healthy aging among older people who are single and among those who live alone.

### The Importance of Economic Status and Perceived Meaningfulness in Life

The differences in perceived health and healthy aging by income level were studied. The participants with the highest monthly incomes had significantly higher HAI scores. This finding is supported by Çapik and Bahar (2008), who find that people with high monthly incomes had positive health perceptions. The current findings may be due to older people with a source of income who might in turn feel financially independent and have access to quality resources and health services. A few participants continued to work after retirement, and a few continued to receive minimal salaries after retirement. Therefore, it was difficult for them to access the services that they wanted on a limited income. Furthermore, older persons are generally likely to be poorer than other members of the population in Thailand (World Bank 2012). Although Thailand has a government-supported monthly pension, it provides little in terms of monetary payments and may not cover necessary expenses for older people. This may affect the perceived health of aging people and should be a focus of policy makers. For example, other sources of incomes for older people unable to work could be supported, and those who are able to keep working after retirement should be encouraged to do so.

Older people who had a high to very high perceived meaningfulness in life had high HAI scores. This finding is in line with a study conducted in Hungary (Skrabski et al. 2005). One explanation for this result may be that perceived meaningfulness in life is associated with positive psychosocial factors (Stillman et al. 2009), which in turn may be related to perceived health (Skrabski et al. 2005). Meaningfulness is the cognitive and emotional assessment that a person has of their life, which is related to value and purpose (Baumeister et al. 2013). If people lose their sense of meaning in life, this may have negative effects on their health (Trice 1990). Based on the present results, it is recommended that older people who reported low to moderate perceived meaningfulness in life be given special attention when promoting healthy aging. Although the findings revealed that older people who had low to moderate perceived meaningfulness in life had low HAI scores, there are remaining questions regarding the implications of the current findings. For instance, if the meaningfulness in life is important for perceived health and health aging, what are the most important factors that make older persons’ lives meaningful? Further, which activities contribute to purpose in older peoples’ lives? Again, the suggestion based on the current findings is linked to the Isan culture in which older people are highly respected in the society (Choowattanapakorn 1999). Older people are supposed to have a wealth of experience, wisdom, and value. Health promotion activities should make older people feel valuable and keep them socially active in their communities. As Pincharoen and Congdon (2003) noted, religious activities are a potent source of meaning in life among older Thai people. Therefore, activities based on the doctrines of Buddhism could be applied to promote healthy aging among people who follow the Buddhist religion.

### Directions for Future Research on Healthy Aging in the Isan Region

The cross-sectional design of this study allows for the description of associations between several factors and HAI scores, but causal conclusions cannot be drawn. A longitudinal study could investigate which factors predict healthy aging. Qualitative studies are required to deepen our understanding of factors related to healthy aging in this region. Older persons with psychiatric problems were not included in this study and should be included in future research regarding healthy aging. Regarding the measurements, there are other instruments might be used. Although the HAI is an appropriate instrument to measure perceived health and healthy aging among older persons, future research should include measurements of functional disability, social network, and Activities of Daily Living (Garcia and McCarthy 1996).

## Conclusions

The promotion of health and maintenance of a sense of well-being among older adults in northeastern Thailand are vitally important aims for policy makers and healthcare providers because the older population is increasing dramatically. The present study revealed differences and similarities between Western and Asian countries in terms of the effects of socio-demographic factors, health status, and perceived meaningfulness in life on perceived health and healthy aging. Understanding these factors should be a starting point for considering healthy aging in northeastern Thailand. Furthermore, the present study is the first phase in identifying the most important determinants of perceived health and healthy aging. In fact, although the final model explained just 24.30 % of the variance in HAI scores, the current findings contribute to our understanding of how area of residence, disability and marital status are associated with perceived health and healthy aging in northeastern Thailand. This study shed light on the key factors that healthcare providers or researchers should consider in intervention studies and programs to promote healthy aging in this region.

## References

[CR1] Ahmad, K., Jafar, T. H. & Chaturvedi, N. (2005). Self-rated health in Pakistan: results of a national health survey. BMC Public Health doi:10.1186/1471-2458-5-51 51. 10.1186/1471-2458-5-51 PMC1164420 15943882

[CR2] Baumeister RF, Vohs KD, Aaker JL, Garbinsky EN (2013). Some key differences between a happy life and a meaningful life. The Journal of Positive Psychology.

[CR3] Benyamini Y, Idler EL, Leventhal H, Leventhal EA (2000). Positive affect and function as influences on self-assessments of health expanding our view beyond illness and disability. The Journals of Gerontology Series B: Psychological Sciences and Social Sciences.

[CR4] Caffrey RA (1992). Caregiving to the elderly in Northeast Thailand. Journal of Cross-Cultural Gerontology.

[CR5] Çapik C, Bahar Z (2008). ). Determination of factors influencing perceived health status among poor and non-poor women in eastern Turkey. International Journal of Caring Sciences.

[CR6] Choowattanapakorn T (1999). The social situation in Thailand: the impact on elderly people. International Journal of Nursing Practice.

[CR7] Cott CA, Gignac MAA, Badley EM (1999). Determinants of self-rated health for Canadians with chronic disease and disability. Journal of Epidemiology and Community Health.

[CR8] Danyuthasilpe C, Amnatsatsue K, Tanasugarn C, Kerdmongkol P, Steckler AB (2009). Ways of healthy aging: a case study of elderly people in a northern Thai village. Health Promotion International.

[CR9] Ekasingh B, Sungkapitux C, Kitchaicharoen J, Suebpongsang P (2007). *Competitive commercial agriculture in the northeast of Thailand. background paper for the Competitive Commercial Agriculture in sub-saharan Africa (CCAA) Study*.

[CR10] Faresjö T, Rahmqvist M (2010). Educational level is a crucial factor for good perceived health in the local community. Scandinavian Journal of Public Health.

[CR11] Fry G, Kempner K (1996). A subnational perspective for comparative research: education and development in Northeast Brazil and Northeast Thailand. Comparative Education.

[CR12] Fujioka R, Thangphet S (2009). Decent work for older persons in Thailand. (ILO Asia-Pacific Working Paper Series)..

[CR13] Garcia P, McCarthy M (1996). *Measuring health.* A step in development of city health profiles.

[CR14] Goldman N, Korenman S, Weinstein R (1995). Marital status and health among the elderly. Social Science & Medicine.

[CR15] Gurung RA, Taylor SE, Seeman TE (2003). Accounting for changes in social support among married older adults: insights from the MacArthur studies of successful aging. Psychology and Aging.

[CR16] Hayashi Y (2003). Practical Buddhism among the Thai-Lao: religion in the making of a region.

[CR17] Institute for population and Social Research Mahidol University. (2014). *Population aging in Thailand* 2014*.* Retrieved from http://www.m-society.go.th/article_attach/12533/16843.pdf.

[CR18] Jang Y, Kim G, Chiriboga DA (2006). Health perception and depressive symptoms among older Korean Americans. Journal of Cross-Cultural Gerontology.

[CR19] Jitapunkul S, Wivatvanit S (2009). National policies and programs for the aging population in Thailand. Ageing International.

[CR20] Kalab M (1990). Buddhism and emotional support for elderly people. Journal of Cross-Cultural Gerontology.

[CR21] Keyes CF (1984). Mother or mistress but never a monk: Buddhist notions of female gender in rural Thailand. American Ethnologist.

[CR22] Klunklin A, Greenwood J, in Thailand AIDS (2005). Buddhism, the status of women and the spread of HIV/. Health Care for Women International.

[CR23] Knodel J, Chayovan N (2008). *Population ageing and the well-being of older persons in Thailand* (Report No. 08–659):.

[CR24] Knodel, J., Prachuabmoh, V. & Chayovan, N. (2013). *The changing well-being of Thai elderly: An update from the 2011 survey of older persons in Thailand*. Chiang Mai: HelpAge International.

[CR25] Knodel J, Teerawichitchainan BP, Prachuabmoh V, Pothisiri W (2015). *The situation of Thailand’s older population: An update based on the 2014 Survey of Older Persons in Thailand*.

[CR26] Kutanan W, Ghirotto S, Bertorelle G, Srithawong S, Srithongdaeng K, Pontham N, Kangwanpong D (2014). Geography has more influence than language on maternal genetic structure of various northeastern Thai ethnicities. Journal of Human Genetics.

[CR27] Lawalee B, Yodmalee B, Champadaeng S (2014). Boon Phawet: A comparative study of a Phu Tai, Tai-Lao and Kaleung religious ceremony in North-eastern Thailand. Asian Culture and History.

[CR28] Molarius A, Janson S (2002). Self-rated health, chronic diseases, and symptoms among middle-aged and older people men and women. Journal of Clinical Epidemiology.

[CR29] Mongkolsawat, C., Thirangoon, P., Suwanweramtorn, R., Karladee, N., Paiboonsank, S. & Champathet, P. (2001). An evaluation of drought risk area in northeast Thailand using remotely sensed data and GIS. *Asian Journal of Geoinformatics*, 1: 33–44.

[CR30] Monnat, S.M. & Pickett, C.B. (2011). Rural/ Urban differences in self-rated health: examining the roles of country size and metropolitan adjacency. Health *& Place*, 17: 311–319. 10.1016/j.healthplace.2010.11.008 21159541

[CR31] Palner J, Mittelmark MB (2002). Differences between married and unmarried men and women in the relationship between perceived physical health and perceived mental health. Norwegian Journal of Epidemiology.

[CR32] Phuttharak T, Dhiravisit A (2014). Rapid urbanization-its impact on sustainable development: A case Study of Udon Thani, Thailand. Asian Social Science.

[CR33] Pincharoen S, Congdon JG (2003). Spirituality and health in older Thai persons in the United States. Western Journal of Nursing Research.

[CR34] Ratanakul P (2007). The dynamics of tradition and change in Theravada Buddhism. The Journal of Religion and Culture.

[CR35] Ren XS (1997). Marital status and quality of relationships: The impact on health perception. Social Science & Medicine.

[CR36] Rukwong P (2008). Lived experience of middle-aged women living with a disability in Isaan, Thailand. Nursing and Health Sciences.

[CR37] Sansak K, Lamduan S, Champadaeng S (2014). Traditional Isan literature and its influence on everyday society. Asian Culture and History.

[CR38] Sasat S, Bowers BJ (2013). Spotlight Thailand. The Gerontologist.

[CR39] Singh, L., Arokiasamy, P., Singh, P.K. & Rai, R.K. (2013). Determinants of gender differences in self-rated health among older population: evidence from India. SAGE *Open.* doi: 10.1177/2158244013487914.

[CR40] Skrabski A, Kopp M, Rózsa S, Réthelyi J, Rahe RH (2005). Life meaning: an important correlate of health in the Hungarian population. International Journal of Behavioral Medicine.

[CR41] Statistical Package of the Social Sciences. (2007). SPSS 17.0. SPSS Inc. Headquaters, Chicago, Illinois 60606.

[CR42] Stillman TF, Baumeister RF, Lambert NM, Crescioni AW, DeWall CN, Fincham FD (2009). Alone and without purpose: life loses meaning following social exclusion. Journal of Experimental Social Psychology.

[CR43] Sudnongbua S, LaGrow S, Boddy J (2010). Feelings of abandonment and quality of life among older persons in rural Northeast Thailand. Journal of Cross-Cultural Gerontology.

[CR44] Suwanrada, W. (2012). *Old-age allowance system in Thailand.* Retrieved from http://www.ipc-undp.org/conference/south-south-learning event/presentations/Worawet%20Suwanrada.pdf

[CR45] Thanakwang K (2009). Social relationships influencing positive perceived health among Thai older persons: a secondary data analysis using the national elderly survey. Nursing and Health Sciences.

[CR46] Thanakwang K, Soonthorndhada K, Mongkolprasoet J (2012). Perspectives on healthy aging among Thai elderly: A qualitative study. Nursing & Health Sciences.

[CR47] Thiamwong (2008). Development and psychometric testing of the healthy aging instrument. (Unpublished doctoral’s desertation).

[CR48] Thiamwong L, Maneesriwongul W, Malathurm P, Jitapunkul S, Vorapongsathorn T, Stewart AL (2008). Development and psychometric testing of the healthy aging instrument. Thai Journal of Nursing Research.

[CR49] Thiamwong L, McManus MS, Suwanno J (2013). Development of the Thai healthy aging model: A grounded theory study. Nursing & Health Sciences.

[CR50] Trice LB (1990). Meaningful life experience to the elderly. Image: The Journal of Nursing Scholarship.

[CR51] Undén A-L, Elofsson S, Andréasson A, Hillered E, Eriksson I, Brismar K (2008). Gender differences in self-rated health, quality of life, quality of care, and metabolic control in patients with diabetes. Gender Medicine.

[CR52] UNFPA. (2006). Population ageing in Thailand prognosis and policy response. Thailand: UNFPA Thailand. Retrieved from http://www.globalaging.org/elderrights/world/2006/thailand.pdf

[CR53] United Nations (2015). *World population prospects*: The 2015 revision.

[CR54] Wanlu S, Chantachon S, Rachote B (2009). An application of Isan local indigenous knowledge in suppression of social disputes. The Social Sciences.

[CR55] Wen M, Hawkley LC, Cacioppo JT (2006). Objective and perceived neighborhood environment, individual SES and psychosocial factors, and self-rated health: An analysis of older adults in Cook Country, Illinois. Social Science & Medicine.

[CR56] Westaway MS (2010). The impact of chronic diseases on the health and well-being of south Africans in early and later old age. Archives of Gerontology and Geriatrics.

[CR57] Williamson, C. (2015). *Policy Mapping on Ageing in Asia and the Pacific: Analytical Report*: Chiang Mai: HelpAge International, East Asia/Pacific Regional Office.

[CR58] Wongtham PS, Chantachon S, Laoakka S (2015). Monk development experts: Using traditional knowledge to manage community development by monks in Isan. Asia Pacific Journal of Multidisciplinary Research.

[CR59] World Bank (2005). *Thailand—Northeast economic development report*.

[CR60] World Bank. (2015). Life expectancy at birth, total (years). Retrieved from http://data.worldbank.org/indicator/SP.DYN.LE00.IN.

[CR61] World Bank. (2012). Reducing older people poverty in Thailand: the role of Thailand’s pension and social assistance programs. The World Bank Office, Bangkok: Thailand.

[CR62] Yiengprugsawan, V., Lim, L. L., Carmichael, G. A., Sidorenko, A. & Sleigh, A. C. (2007). Measuring and decomposing inequity in self-reported morbidity and self-assessed health in Thailand. International Journal for Equity in Health. doi:10.1186/1475-9276-6-23 10.1186/1475-9276-6-23 PMC2242789 18088434

[CR63] Zunzunegui MV, Kone A, Johri M, Beland F, Wolfson C, Bergman H (2004). Social networks and self-rated health in two France-speaking Canadian community dwelling populations over 65. Social Science & Medicine.

